# A narrative literature review to inform the development of a health threats preparedness framework in Ireland

**DOI:** 10.3389/fpubh.2025.1490850

**Published:** 2025-02-06

**Authors:** Louise Marron, James Gilroy, Michelle Williams, Randal Parlour, Máirín Boland

**Affiliations:** ^1^Health Service Executive-Health Protection Surveillance Centre, HSE National Health Protection Office, Dublin, Ireland; ^2^National Health Security/ Threats Preparedness Programme, HSE National Health Protection Office, Dublin, Ireland; ^3^Research & Guideline Development Unit, HSE National Health Protection Office, Dublin, Ireland; ^4^UCD School of Public Health, Physiotherapy and Sports Science, University College Dublin, Dublin, Ireland

**Keywords:** preparedness, health threats, all-hazards, pandemic planning, emergency planning, one health, health security

## Abstract

**Introduction:**

Public health emergency preparedness requires capacities and capabilities to respond to a diverse range of health threats. A key objective of Ireland’s recent Health Protection Strategy is to enable preparedness, prevention, early detection and optimal response to health threats from all-hazards. We aimed to identify priority areas for inclusion in an evidence-based health threats preparedness framework, using lessons from the COVID-19 pandemic, to inform a national health threats strategy and a strengthened emerging health threats function for Ireland.

**Methods:**

We conducted a narrative literature review to inform the health threats preparedness framework development. We carried out literature searches in two phases, from 2017 to 2022, followed by an updated search covering 2022–2024, to ensure all relevant, recent literature was captured. We used a data extraction tool to collate priority areas reported.

**Results:**

Overarching priorities for health threats preparedness are rapid decision-making, an outcomes-based, and ethical approach. Health threats preparedness should include a risk-based, all-hazards, One Health approach, aligned with legislation. Multisectoral partnerships, collaboration and communication nationally and internationally are key, alongside clear governance structures and monitoring and evaluation. Adequate resources are required to operationalize effective and sustainable preparedness. Public health leadership must be to the fore.

**Conclusion:**

An effective health threats preparedness approach is legislatively mandated for European Member States. This evidence review highlights priority areas for a comprehensive health threats preparedness framework. This framework supports the development of a strengthened emerging health threats function in Ireland and may inform other Member States’ preparedness.

## Introduction

Arising from the COVID-19 pandemic, a new legislative architecture has strengthened European Union (EU) Member States’ (MS) preparedness and operational readiness for future health crises (1). Public health emergency preparedness (PHEP) is the capability of the public health and healthcare systems, working with others, to prevent, protect against, quickly respond to and recover from health threats and emergencies (2). A public health threat is an event or condition arising from an agent (hazard) with the potential to rapidly harm an exposed population and cause a crisis (3). PHEP is a coordinated and continuous cycle of planning, implementation, measuring performance and taking corrective action, and requires both capacities and capabilities (4).

In Ireland, a new National Health Protection function was established in 2022, as part of a process of public health medicine reform (5). This function takes an all-hazards approach with programmes across surveillance, health security, response and immunization, (6) and it aligns with national health system-wide reform which emphasizes public health and prevention (7). A key objective of Ireland’s recent National Health Protection Strategy (2022–2027) is to enable preparedness, prevention, early detection and optimal response to public health emergencies from all-hazards (5, 8).

A national Public Health Reform Expert Advisory Group reported in September 2023 on Ireland’s public health response to the COVID-19 pandemic (9). An independent expert review commenced in December 2023 to design a national dedicated emerging health threats function and the proposal report was published in October 2024 (10).

We aimed to identify priority areas for inclusion in an evidence-based health threats preparedness framework, informed by learnings from the COVID-19 pandemic; to inform an emerging health threats strategy and function for Ireland; and to contribute to other countries’ work in this area.

## Methods

We undertook a narrative literature review to identify the components of an effective health threats preparedness framework. We identified evolving themes and priority areas in health threats preparedness, including those informed by the COVID-19 pandemic, to inform health threat preparedness framework and strategy development.

### Information sources and search phases

Search terms were identified by pearl-growing from seminal papers in the field, guided by the consensus of relevant content experts. Using the assistance of an information specialist, we refined our search approach to be specific, capturing literature covering health threats, preparedness/response approach and including a global or cross-border element. The search terms used are included as Box 1. Peer-reviewed and grey literature publications were identified by hand-searching electronic databases, PubMed and Google Scholar, including use of the advanced search feature. A librarian-curated feed (provided by the Health Service Executive (HSE) Library) was also available, which identified relevant papers for inclusion. Additional publications were identified by citation chasing. Targeted hand-searching of the World Health Organization (WHO) and the European Centre for Disease Prevention and Control (ECDC) websites was carried out to identify key strategy and framework documents for inclusion in this review. The initial search covered January 2017 to June 2022 (Phase 1), to develop an initial health threats preparedness framework and was conducted in July 2022, for the launch of Ireland’s first National Health Protection Strategy [5]. The search was updated in February 2024 to cover July 2022 to January 2024 (Phase 2). This was to ensure that relevant and up-to-date literature was captured to inform framework development, including post-pandemic lessons learned.

BOX 1 **Search terms**

Health threats, health protection threats, communicable disease threats, health threats preparedness, health hazards, public health emergency preparedness and/or planning, response to all hazards, pandemic preparedness, outbreak preparedness and/or response, chemical, biological, radiological and nuclear (CBRN) threats, health security, International Health Regulations (IHR), cross-border health security, port health, international, operational, model, structure, programme.

### Eligibility criteria

Publications pertaining to health threats preparedness, particularly those presented through a programmatic or strategic lens, were included in this review. These included peer-reviewed publications and relevant grey literature documents published on organizational websites. Grey literature documents included in this review comprised strategy documents, action plans, frameworks, policy briefs, reports published nationally or internationally, and documents published by international organizations. Documents or publications covering only specific non-communicable health threats were excluded.

### Data extraction

Identified full texts underwent single stage screening by one person. Data extraction was undertaken by two researchers in both phases. All outputs were appraised and validated by at least two independent researchers. Findings reported in the included full texts were extracted using a data extraction tool. This tool recorded the author, year of publication, a summary of the key points reported by the authors and the relevant strategic objectives for health threats preparedness that were stated in the document. When extracting data from the full texts included in this review, we employed the steps of two reviewers’ initial familiarization with the full text, thematic categorization, theme review and consensus thematic definition by all authors to achieve a summary of priority key points and relevant strategic objectives for health threats preparedness. Data were collated and compiled under the headings reported in this literature review reflecting those priority areas. A table of included documents is included as Supplementary Table S1.

## Results

### Study selection

**Figure 1:**
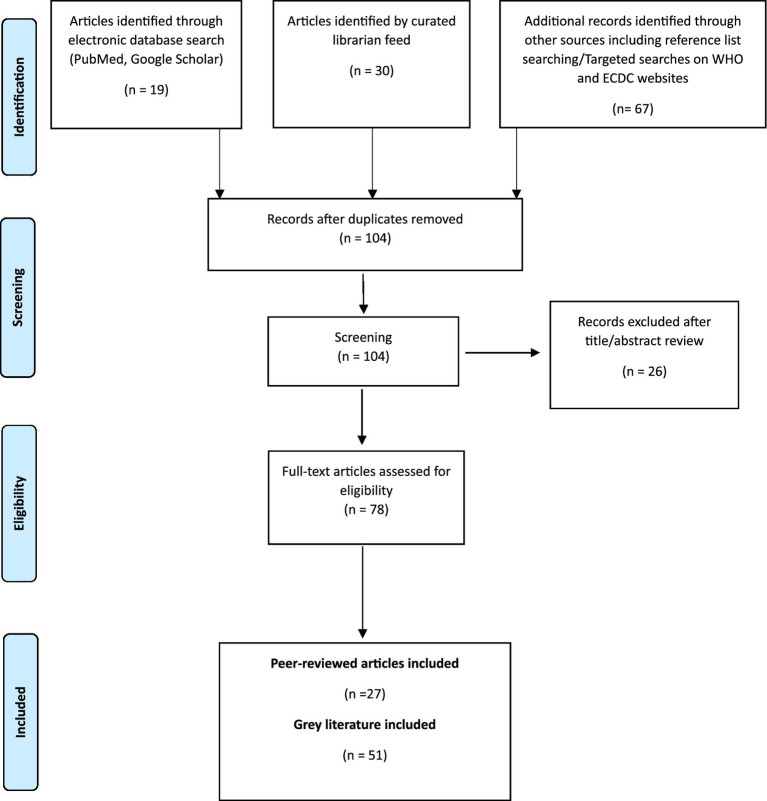
Flow diagram of literature search.

The literature search yielded 116 articles and following removal of duplicates and title, abstract and full text review, 78 articles were included; 27 articles from electronic database searches, and 51 grey literature articles (Figure 1).

### Priority components for a health threats preparedness framework

**Figure 2:**
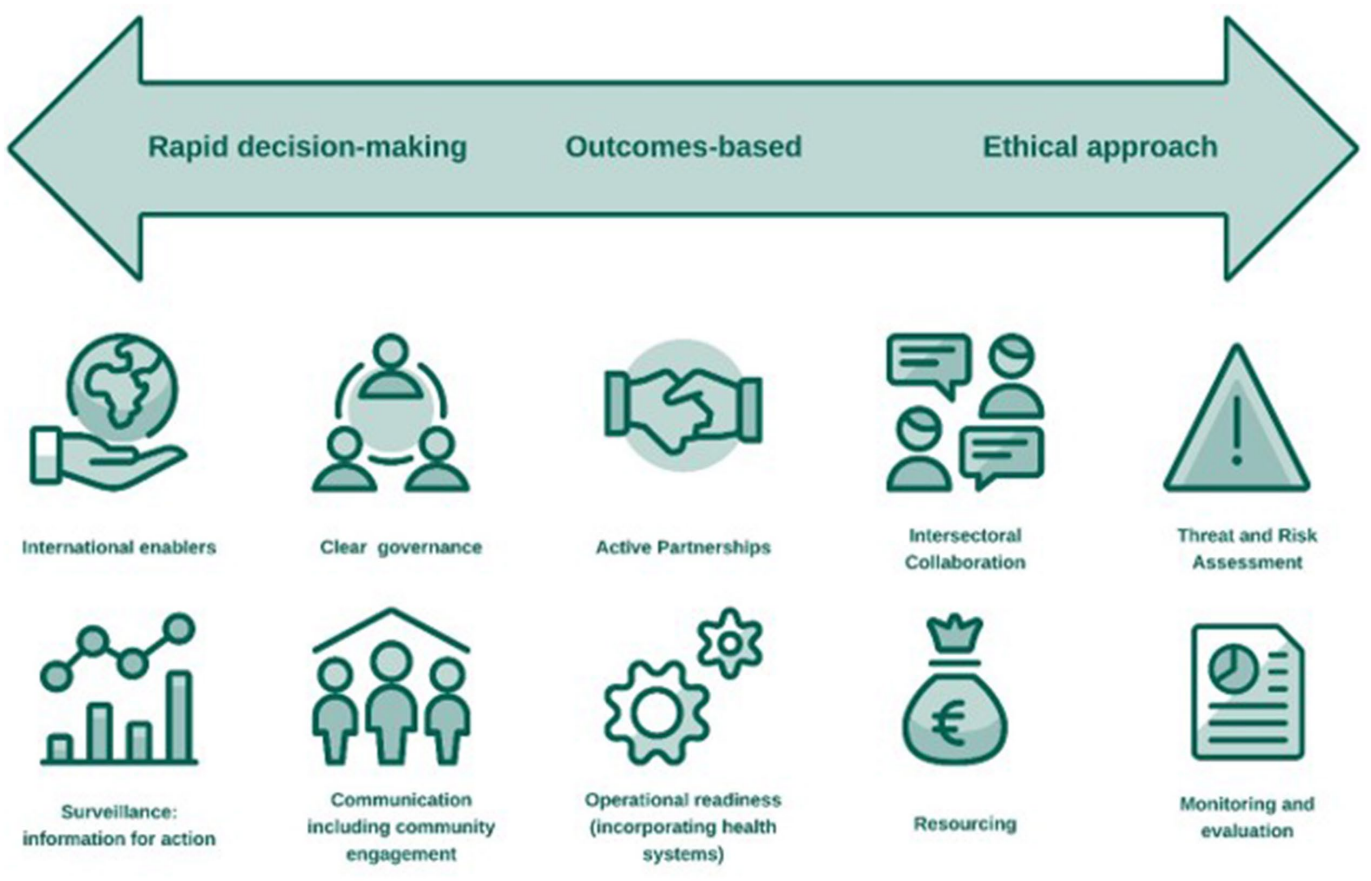
Health threats preparedness framework consisting of three overarching priorities and ten key components.

We report our findings in the context of recent shifts in EU policy and legislation since the onset of the COVID-19 pandemic to strengthen countries’ readiness for serious cross border threats to health. From the literature we propose a health threats preparedness framework with three overarching priorities and 10 key components (Figure 2).

### Overarching priorities

#### Rapid decision-making

The COVID-19 pandemic highlighted the need for rapid decision-making in response to health threats (11, 12). Public health decision-making at local, national, regional and global levels must be based on real-time, accurate data (13, 14). Rapid decision-making therefore requires prompt identification of threats followed by evidence-based, data-driven, inclusive decision-making to guide timely response (11). Recently strengthened EU legislation is a key enabler, emphasizing the need for early warning systems, partnership with key stakeholders to approach decisions from a One Health approach and joint procurement of medical countermeasures (15). The establishment of the Health Emergency Preparedness and Response Authority (HERA) will enable access to medical countermeasures in a timely manner (16, 17). Shared analysis of threats and jointly agreed priorities for action will strengthen rapid decision-making, with community empowerment key (16, 18).

#### Outcomes-based

Measuring the components of emergency preparedness is challenging (19). The COVID-19 pandemic identified limitations in how health threats preparedness is measured (11, 20, 21). The pandemic highlighted how markers of preparedness, e.g., measuring capacities, did not correlate with pandemic outcomes (22). Achieving core capacities may not adequately prepare countries for a high-impact health threat (23). An outcomes-based approach has been proposed as an alternative to traditional capacity assessments (21). This approach would evaluate the implementation of interventions and their outcomes and may be more beneficial in identifying preparedness weaknesses (21). Further work is required to develop and validate indicators for relevant outcomes (22). It has been recognized that preparedness assessment should consider contextual factors in which emergencies occur, such as the sociopolitical environment, which may influence outcomes (21).

#### Ethical approach

Ethical principles and values are central to public health practice (24). Values important to health threats preparedness include equity, trust, public protection, duty of care and solidarity (12, 24–26). These considerations are important in the context of limited resources, challenges with access to medical countermeasures and underlying issues with public trust (24). The COVID-19 pandemic highlighted inequity and the need for an inclusive, equitable approach to preparedness and response (17, 20, 26–28). The pandemic also emphasized the need for information to be communicated accurately, consistently and coherently (11). The operationalization of an ethical approach involves anticipating risks and undertaking preparedness activities specific to relevant, vulnerable populations. These specific activities should inform the development of strategic objectives, priority actions and goals, recognizing vulnerabilities of specific population groups, such as children and those in minority groups (29–32). There is no clear method of monitoring the application of an ethical approach to health threats preparedness reported in the literature.

### Ten key components

#### International enablers

The new European Health Union coordinates EU preparedness activities (33). The 2021 establishment of HERA with its focus on stockpiling, medical countermeasures and data flow, has improved the ability of health systems to respond to health threats in a coordinated manner (16, 18). The EU4Health programme reinforces crisis preparedness, and the strengthened mandate of the ECDC and the Health Security Committee provide critical support for countries in threat preparedness and response (17, 33, 34).

Updated international legislation and regulations emphasize prevention and strengthening of health security (12, 15, 17). The EU Regulation on serious cross border threats to health (2022/2371) covers areas such as cross-EU joint procurement for medical countermeasures, strengthening surveillance activities and establishing EU reference laboratories; key enablers for national threats functions (15). The Regulation aligns with priorities identified in the literature as core components for health threats preparedness, including interoperability, collaboration and an all-hazards approach (17, 22, 24, 35, 36).

#### Clear governance

Clear governance structures are necessary both at EU member state level and cross-border, alongside public health and political leadership, promoting a culture of preparedness (31, 37). Integration of public health with health and non-health sectors, with explicit leadership and partnership with appropriate accountability, is essential for a coordinated, interoperable, cross-sectoral approach (13, 24, 38). The COVID-19 pandemic highlighted he imperative to improve health governance globally (39, 40).

Understanding and defining where public health sits within governance structures is key; requiring clarity in the identification of principal agencies and authorities, especially in response to non-infectious health threats (13, 24, 41). Establishing roles and responsibilities of stakeholders, with clear governance structures is crucial for preparedness (3, 11, 41, 42).

#### Active partnerships

A partnership approach to health threats preparedness is emphasized in legislation (12, 15). Preparedness is not an exclusive function of health sectors; it is a shared responsibility requiring a whole-of-government, whole-of-society approach (13, 41, 43–45). Stakeholder mapping and prioritization, strong relationships and partnerships between government sectors, health sectors, commercial sectors, communities and within society can achieve collective preparedness (13, 15, 24, 35, 39, 43–47).

There are increasing risk factors for health emergencies, including climate change and environmental degradation (13, 20, 36, 48–51). Threats to health security can rapidly affect multiple countries highlighting the need for collective preparedness and for more effective international collaboration, active partnerships and common strategic plans (12, 15, 41).

#### Intersectoral collaboration

Effective intersectoral and multisectoral collaboration in health threats preparedness remains a challenge (31, 41, 52). Health threats preparedness frameworks should include a One Health and all-hazards approach, with support of hazard-specific plans (21, 22).

##### One health approach

Emerging zoonotic threats and antimicrobial resistance (AMR) threaten both human and animal health, necessitating a One Health approach (35, 45, 52, 53). Responding to the challenges of emerging and re-emerging infections requires strengthening of surveillance, risk assessment and laboratory capacity, and risk communication (35, 47). Information sharing and co-operation between human and animal health services are essential (13, 43). A key lesson from the COVID-19 pandemic is that the concept of One Health should be operationalized at all levels and a One Health approach to disease surveillance should be incorporated into preparedness (11, 21, 28, 39). Countries should invest in One Health preparedness and focus on developing multisectoral and intersectoral collaboration (21, 54).

##### All-hazards approach, complemented by hazard-specific measures

An all-hazards approach recognizes that risks to human health can emerge from diverse sources, including infections transmitted via goods, food, water or animals as well as chemical, radiation, nuclear and environmental events (30–32, 46, 52). Chemical, biological, radiological and nuclear (CBRN) refers to categories of materials and agents that could harm society due to their accidental or deliberate release, dissemination or impacts (43, 55, 56). CBRN incidents require specific preparedness and response (32, 44, 55, 57, 58). Many elements of preparedness are common to all-hazards (3, 44, 59). Therefore, a common, coordinated intersectoral approach, comprising both all-hazard and hazard-specific measures and capabilities, is required and is mandated by legislation (15, 23, 35, 36, 46, 60).

#### Threat and risk assessment

All-hazard risk-mapping is required to inform health threat preparedness (31, 52). Identifying and prioritizing threats and the capabilities required to prepare and respond is essential (60, 61). Risk assessments can be undertaken using tools developed by WHO, e.g., the Strategic Toolkit for Assessing Risks (STAR) or the risk communication and community engagement tool (3, 22, 62). WHO benchmarks for strengthening emergency capacities include having established rapid risk assessment processes linked to response plans (63).

While WHO guidance has been designed to be adapted to individual country situations, capacities and requirements (3), there has been a lack of a common methodology used by countries for assessing risk, as well as different perceptions as to what risks are; leading to different policies and different levels of preparedness and response capabilities (55, 56, 61, 64, 65). The need for rationalizing and coordinating risk assessment frameworks incorporating learnings from the COVID-19 pandemic, while allowing countries to identify relevant hazards has been recognized (21, 41).

#### Surveillance: information for action

Robust and accurate surveillance data are crucial to health threats preparedness, particularly identification of novel pathogens (15, 66, 67). National surveillance systems must be capable of timely detection, assessment and analyses of epidemiological data, including laboratory results, for informed decision-making and reporting of outbreaks and other public health risks (15, 31, 46, 51, 68). Surveillance systems should include indicator and event-based surveillance and should adopt a One Health, all-hazards approach (15, 22, 41, 55, 56, 69, 70). Surveillance must be supported by digital platforms and by integrated, efficient, effective and timely early warning systems, particularly for priority hazards (17, 31, 41, 67, 68). Notification to early warning systems should be supported by having updated contact points and standard operating procedures (4). Data sharing procedures across sectors and regionally and nationally should be strengthened to enable collaborative surveillance (31, 39). Genomic surveillance expanded during the COVID-19 pandemic and is important to strengthen surveillance and increase capacity to detect health threats (71). Genomic data can inform risk assessment, development of medical countermeasures and public health decision-making (72).

#### Communication including community engagement

Communication is dependent on partnership and collaboration within and outside health sectors (13, 24). Intersectoral risk communication should communicate health threats in a timely, coordinated and transparent manner (3, 31, 55) to enable decision-makers, stakeholders and the public to make informed and appropriate decisions. Risk communication requires adequate resources and multiple communication modalities (4, 35). An all-hazard emergency risk communication function should be integrated into national action plans for emergency preparedness (15, 56). The COVID-19 pandemic highlighted the complexity of communication in a pandemic and the importance of accurate, scientific, expert-led, risk communication during a pandemic (41, 73, 74). It also highlighted communication challenges in the context of scientific uncertainty, an abundance of information, and infodemic management (20, 73, 75).

Community engagement is essential for effective risk communication and preparedness (4, 35, 41, 43, 68, 74). Active, two-way engagement is key to understanding risk perception and identifying and addressing myths and disinformation (53). Communication campaigns for health emergencies should be grounded in behavioral science, and should consider cultural contexts and inclusive language for all populations (20, 53). Investment is key before, during and after health emergencies to enable individuals, families and communities to engage in preparedness (20, 28, 76).

#### Operational readiness (incorporating health systems)

Achieving operational readiness involves establishing, strengthening and maintaining a multisectoral response infrastructure which focuses on the highest priority all-hazard risks (13, 38, 62). This requires political commitment, coordination, risk assessment, infrastructure, preparedness plans, resources, training, and expert knowledge (13). Health emergencies weaken health systems and weak health systems worsen health emergencies (30, 31). Preparedness is part of health system resilience (77). However, building resilience in health systems is challenging (78). Robust, sustainable and accessible health systems are essential for health threats preparedness (3, 4, 12). There is a need to strengthen health systems to support preparedness (12, 37). Health system response requires rapid risk assessment, testing, diagnostics, contact tracing, clinical evaluation and care, surge workforce and rapidly scalable interventions such as timely procurement and capacity and capability to distribute medical countermeasures (13, 36, 37, 41, 47, 52, 79).

The COVID-19 pandemic exposed vulnerabilities of national health systems and identified the need for targeted preparedness planning and long-term investment in strengthening health systems, including workforce (11, 41). Essential procurement should be informed by national risk profiles, and supply chain management systems should be strengthened to ensure operational readiness (4, 13, 31). A pre-negotiated platform for medical countermeasures would ensure rapid and equitable delivery globally (11, 20, 41, 80).

#### Resourcing

The COVID-19 pandemic highlighted the need for further financial investment to reduce threats, provide early warning systems and improve capacity to respond to crises (11, 40, 41, 47). Additionally, investing in the highly skilled workforce required for preparedness is essential (28, 31, 41, 70). Investment in preparedness for population health and health security must be sustainable and maintained during the period between emergencies (13). WHO recommend that resources should be integrated into national budgets and planning cycles (35, 52). Adequate resourcing includes investment in facilities, including health facilities, and other infrastructure such as laboratory testing capacities, healthcare surge capacity and management of medical countermeasures (2, 11, 19, 81). Necessary infrastructure and capacity for mobilizing resources and activating preparedness plans should be resourced, and priorities should be established for allocation of limited resources (24).

Dedicated resources should be made available to support an active research and evidence synthesis function. This should include research development and evaluations to inform and accelerate evidence-informed emergency preparedness at all levels (15, 41).

#### Monitoring and evaluation

Health threats preparedness plans should be updated, reviewed and tested to ensure that adequate capacity for effective preparedness and response is developed, maintained and strengthened (28). Ongoing monitoring and evaluation facilitate continuous learning for quality improvement, through reviewing experiences and incorporating lessons learned (28, 35). Examples of assessments include external evaluations, self-assessment tools, simulation exercises and after-action reviews detailed in the IHR monitoring and evaluation framework (IHRMEF) and the periodic assessments described in Article 8 of the European Regulations (15, 30, 31, 65, 82–85). The need for enhanced preparedness monitoring was a key learning from the COVID-19 pandemic (41, 49). The pandemic highlighted the need to shift focus to an outcomes-based approach for measuring preparedness (21). Ongoing work is required to determine how to effectively monitor preparedness (17).

### Summary of findings

This literature review has identified key components for inclusion in a health threats framework. Our proposed framework suggests a model that can be used and adapted to inform health threats preparedness programmes. A summary of the guiding principles to aid development of a health threats framework is in Supplementary Table S2.

## Discussion

The literature describes the strong international enablers for health threats preparedness which have been established since COVID-19, both legislative and structural (15). The European Health Union including HERA (16), a stronger European Medicines Agency and a more influential ECDC with increased remit of the Health Security Committee provides a strong basis to support MS threats strategy development. Commentators have discussed the interplay between European stakeholders and MS and the challenge to further clarify roles and responsibilities to avoid ambiguity, overlaps and gaps (16). Member States’ (MS) national threats functions require clear lines of governance within and external to EU health security structures, including in the areas of horizon scanning, threat assessment, procurement and stockpiling, with clarity on joined-up operational implications for MS.

National legislation on emergency response will need to be consistent with updated international legislation. This should encompass legal, administrative and other governmental instruments required to implement the legislation (47, 52) following IHR revision (12), and pandemic treaty negotiations (86). Policy development, adaptation and implementation are core capacities and capabilities to allow national focal points to perform their functions under IHR (2, 12).

Strong public health leadership and governance are described in the literature as critical to ensure resilience, sustainability and accountability to respond immediately to health threats. In Ireland, a Public Health intra-action review of the COVID-19 response found governance to be a leading challenge (87). As with many MS, reform is underway in Ireland; alongside Public Health reform (9) and health service overarching reform (7), it is critical to maintain and further develop our health protection capacities and capabilities to lead on interoperable preparedness and response (5). In Ireland, the strengthening of a health threats function is underway to ensure that the health system is equipped to identify and respond to future crises arising from all hazards. Ongoing reform within our health service and within public health is an opportunity to identify gaps including the need to enhance all hazards surveillance, and strengthen a One Health approach (10).

Active partnerships and intersectoral collaboration are central to any health threats strategy. In addition to much grey literature recently in this area, with a focus on One health and all-hazards, authors discuss the imperative for partnerships in preparedness to enable interoperable response (11, 13, 15, 21, 28, 46). The implication for national frameworks/strategies is to establish partnerships in all key areas, to leverage and build on existing networks, to avoid overlap and bring cohesion, and to plan and exercise scenarios together ahead of any event. A whole-of-government and whole-of-society, national and international approach is critical (13, 41, 43–45). In Ireland, we must continue to identify and focus on vulnerable groups and underserved populations to ensure an equitable and ethical approach to health threats preparedness. This will require collaboration with social and behavioral scientists to tailor consistent and transparent communication before, during and after emergencies.

From the literature other key areas include threat and hazard assessment, informed by surveillance across all-hazards; it will be a new challenge for countries to move beyond the traditional infectious disease focus. Community engagement is critical, particularly for public health social measures (88). Nationally, the health protection function works with key stakeholders including advocacy groups, the HSE national social inclusion office and community representatives. Establishing these collaborative relationships in advance of the next health emergency is crucial and involves co-developing interventions and communications, identifying key influencers and building trust to deliver public health messages.

Literature from mid-2022 onwards critiques the narrow focus pre-COVID within the health system on capacities: the need to build and maintain wider capabilities has been described (60) with proposed frameworks to support ‘health capabilities-based planning’ (examples include capability to provide personal protective equipment (PPE) for staff, capability to undertake waste sterilization) for multiple and diverse threats (60).

Resourcing is often siloed by sector (21), and the imperative for joined-up interoperable preparedness is key, including resourcing of cross-sectoral research activities to guide evidence-based preparedness. In Ireland, the importance of funding and adequate resources for public health, including all aspects of health threats preparedness, has been acknowledged (10). Maintaining focus on the resources required health threats preparedness outside of crisis situations is essential but remains challenging (10).

Post COVID-19, monitoring of preparedness may need to further develop beyond those currently in use such as WHO SPAR (89) and EU Article 8 (15). Some suggest that intercountry comparison is unhelpful (52). The use of matrix models has been suggested, to monitor and assess achievement of outcomes (21). Individual countries should plan, test and exercise in challenging scenarios testing multisectoral interoperability, communications and connectedness, establishing a baseline within a national context and building on this (21).

Extrapolating from this literature review, we propose that a transformation initiative is needed to support the three overarching themes of rapid shared decision-making, outcomes-based approach, using an ethical lens, and to operationalize the 10 key components.

There has been a call for change from other commentators to ‘facilitate the development of thinking towards systems-based, all-hazards frameworks that acknowledge the wider complexities within which public health operates’ with the socio-political context being key (21). ‘Cross disciplinary thinking’ is described in one paper in developing preparedness tools including addressing inequities. Our threats strategy must incorporate preparedness for special population subgroups with different characteristics both in preparedness planning and response scenarios (32), taking account of issues such as vulnerability, shared governance, access, language and cultural norms (20).

Successful transformation requires that external factors must be taken into account: using for example a PESTLE analysis to monitor the macro environment, including megatrends (90). Scenario planning is being used at EU level to test readiness (91, 92). Preparedness plans of all MS will be assessed under EU legislation over the next 3 years (15); sharing of approaches across countries will support European MS to transform towards best practice, with cross-border interoperability.

A national approach to emerging health threats, in both preparedness and response phases, is a reflection of the national environment at both a political and operational health response level. The application of the 10 key components identified across the three overarching priority areas in any given MS would need to be cognizant of the political environment in which a system is operating, and we present here a brief reflection on this interplay in Ireland, to assist readers in applying the findings in their own political context.

Pre-pandemic, Ireland was facing the unique political challenge of Brexit, requiring additional focus on cross border coordination in health threat preparedness and management (93). Post-pandemic, in October 2024 Ireland’s Expert Group on Emerging Health Threats reported with recommendations to strengthen national emerging threat preparedness, building on innovations that occurred during the pandemic (10). Their report reflects the key components of a threat preparedness framework that we found in this literature review, including shared cross-border surveillance, community engagement and active partnership across the two jurisdictions on the island of Ireland (10).

We consider our experience in Ireland regarding the three overarching priorities identified in this literature review: rapid decision-making, outcomes-based approach and ethical approach. Regarding decision-making, one strength in Ireland’s COVID-19 response was a collaborative approach between political leadership, the Department of Health and the Health Service Executive in Ireland, with response centrally coordinated at the highest political level in the country. However, the challenge of governance/accountability and communication across the health system at the onset of this pandemic has been noted; a detailed review of measures for Nursing Home covering the period to end 2021 recommended linked cross-sectoral teams across the community, with rapid communication channels (94).

For the priority area of outcomes, in Ireland excess mortality was comparatively low and vaccination rates were among the highest in Europe (9). Evidence is now accumulating on unintended consequences of COVID-19 response across Ireland and Europe (95–97), which must be considered as we prepare for any future event.

An ethical and values driven approach is more difficult to measure. Ireland’s National Public Health Emergency Team were advised by a Pandemic Ethics Advisory Group (98) but clearly there are numerous aspects of the response to be considered under an ethical spotlight. In Ireland, a review of the COVID-19 response has recently commenced (October 2024) (99), to provide recommendations to strengthen decision-making, to assist in assessing and balancing the complexity of potential trade-offs for decisions, along with the need for speed and agility, and to provide a framework to ensure democratic processes and civil rights are safeguarded in context of whole of society responses to rapidly moving threats (10, 98).

### Limitations

This was a narrative literature review and thus lacks the rigor of a systematic review. Our search was guided by the needs/aims of the review and the subject matter expertise of the team. This approach and the biphasic search strategy may limit the reproducibility of this work. We noted a gap in the literature in relation to country-specific operational structures used in health threats preparedness programmes. Supplementary research, such as qualitative stakeholder consultation, or a targeted scoping review, is required to address this gap. Recent high-level work undertaken, reviewing Public Health systems and structures internationally (100), can inform further development of operational structures focused on all-hazard threats.

## Conclusion

As we emerge from the COVID-19 pandemic, we have an opportunity to reflect and consider how best to prepare for such a future eventuality.

This literature review has informed a framework to contribute towards the development of a national evidence-based health threats strategy. A national strategy must be underpinned by multi-level, multisectoral engagement and a whole-of-society, whole-of-government approach that is built on leadership, partnership, collaboration and coordination both nationally and internationally. It must enable rapid decision-making, have a focus on outcomes for our populations, and be ethically sound. Partnership permeates through much of the literature: we must work together across sectors, collaborating with communities, and across boundaries and borders, developing common understandings. Public health leadership, advocacy and collaboration will be essential to the design and implementation of this evidence-informed initiative; working to prevent and protect our populations against diverse health threats.

Our proposed framework for health threats preparedness contains several elements identified in previous frameworks for health threats preparedness, including an emphasis on governance, collaboration and partnership (24). Moreover, our proposed framework also incorporates learnings arising from the COVID-19 pandemic, nationally and internationally (9, 11) and highlights the importance of strengthening systems to enable rapid decision-making and the imperative of participatory community engagement. Additionally, our framework includes more recently proposed approaches such as an outcome-based approach (90), and places all these priorities in the context of relevant legislative and regulatory frameworks (15).

This framework proposes a comprehensive, up to date and evidence-based approach to health threats preparedness which is being used to progress reform and preparedness nationally and may be of interest to other countries who are strengthening health threats preparedness activities. A resourced strategic transformation initiative, based on evidence and context, will enable Ireland to prepare and respond to future threats within the increasingly complex systems in which health emergencies occur.
