# Supplementary material for: A narrative literature review to inform the development of a health threats preparedness framework in Ireland
Source: Front Public Health. 2025 Feb 6;13:1490850. doi: 10.3389/fpubh.2025.1490850 (PMC11841503; doi:10.3389/fpubh.2025.1490850)
Supplement: Supplementary file 1 [file Data_Sheet_1.docx]

**Supplementary materials**

**Summary of included literature**

**Table S1 Peer reviewed and grey literature included in the literature review**

| **Phase 1 January 2017 to June 2022** | |
| --- | --- |
| **Organisation/Country/Author** | **Title** |
| Ario et al(1) | The logic model for Uganda's health sector preparedness for public health threats and emergencies. Glob Health Action. |
| Australia(2) | Australia’s National Action Plan for Health Security 2019-2023. Implementation of the recommendations from the Joint External Evaluation of IHR Core Capacities. |
| Centers for Disease Control and Prevention(3) | Centers for Disease Control and Prevention Global Health Strategy 2019-2021. |
| Deloitte Centre for Health Solutions(4) | Bridging the gap: Protecting the nation from public health threats. |
| ECDC(5) | ECDC HEPSA Tool. |
| ECDC(6) | ECDC Public Health Emergency Preparedness Core Competencies. |
| ECDC(7) | Health Emergency Preparedness for imported cases of high consequence infectious disease. |
| ECDC(8) | European Centre for Disease Prevention & Control (ECDC) Strategy 2021-2027. |
| ECDC(9) | ECDC tool for the prioritisation of infectious disease threats – Handbook and manual. |
| ECDC(10) | Community engagement for public health events caused by communicable disease threats in the EU/EEA. |
| ECDC(11) | A literature review on community and institutional emergency preparedness synergies. |
| EMA(12) | Plan for emerging health threats. |
| England(13) | Public Health England Policy Paper: Transforming the public health system: reforming the public health system for the challenges of our times, 2021. |
| England(14) | Public Health England Infectious Diseases Strategy 2020-2025. |
| England(15) | Public Health England Strategy 2020-2025. |
| European Commission(16) | Introducing HERA, the European Health Emergency preparedness and Response Authority, the next step towards completing the European Health Union. |
| European Commission(17) | Action Plan to enhance preparedness against chemical, biological, radiological and nuclear security risks. |
| European Parliament(18) | European Union preparedness and response to CBRN threats. |
| European Parliament(19) | Member States’ Preparedness for CBRN Threats. |

**Table S1 Peer reviewed and grey literature included in the literature review (contd)**

| **Organisation/Country/Author** | **Title** |
| --- | --- |
| European Parliament(20) | Regulation (EU) 2021/522 of the European Parliament and of the Council establishing a Programme for the Union's action in the field of health (EU4Health Programme) for the period 2021-2027. |
| Gontariuk et al,(21) | The European Union and Public Health Emergencies: Expert Opinions on the Management of the First Wave of the COVID-19 pandemic and suggestions for future emergencies. |
| Government of Wales(22) | The communicable disease outbreak plan for Wales. |
| Johns Hopkins Center for Health Security(23) | Preparedness for a High-Impact Respiratory Pathogen Pandemic |
| Khan et al,(24) | Public health emergency preparedness: a framework to promote resilience. |
| Pan-European Commission on Health and Sustainable Development(25) | Drawing light from the pandemic: A new strategy for health and sustainable development. |
| Rose et al,(26) | The evolution of public health emergency management as a field of practice. |
| Sharp et al,(27) | Policy and planning for large epidemics and pandemics lessons learned from COVID-19. |
| Stoto et al,(28) | A Public Health Preparedness Logic Model: Assessing Preparedness for Cross-border Threats in the European Region |
| The independent panel for pandemic preparedness and response(29) | COVID 19: Make it the last pandemic. |
| USA(30) | U.S. Department of Health and Human Services National Health Security Strategy 2019-2022. |
| USA(31) | U.S. Department of Health and Human Services Office of the Assistant Secretary for Preparedness and Response: Strategic Plan for 2020-2023. |
| WHO(32) | WHO guidance on preparing for national response to health emergencies and disasters. |
| WHO(33) | WHO Strategic Framework for Emergency Preparedness. |
| WHO(34) | Action plan to improve Public Health Preparedness and Response in WHO European Region. |
| WHO(35) | Asia Pacific Strategy for Emerging Diseases and Public Health Emergencies (APSED III). |
| WHO(36) | WHO Strengthening pandemic preparedness for respiratory pathogens: A policy brief. |
| WHO(37) | WHO emergency response framework. |
| WHO(38) | IHR Monitoring and Evaluation Framework. |
| WHO(39) | NAPHS for all. A 3 step strategic framework for national action plan for health security. |
| WHO(40) | NAPHS for all. A country implementation guide for national action plan for health security. |
| WHO(41) | Strategic Preparedness, Readiness and Response Plan to End the Global COVID-19 Emergency in 2022. |
| WHO(42) | Report of the review committee in the functioning of the International Health Regulations (2005) during the COVID-19 response. |

**Table S1 Peer reviewed and grey literature included in the literature review (contd)**

| **Organisation/Country/Author** | **Title** |
| --- | --- |
| WHO(43) | International Health Regulations (2005). |
| WHO(44) | Global genomic surveillance strategy for pathogens with pandemic and epidemic potential, 2022–2032. |
| Wijesinghe et al,(45) | Pandemic influenza preparedness in the WHO South-East Asia Region: a model for planning regional preparedness for other priority high-threat pathogens. |
| **Phase 2 July 2022 to January 2024** | |
| Alakija et al,(46) | Leveraging lessons from the COVID-19 pandemic to strengthen low-income and middle-income country preparedness for future global health threats. |
| Berg et al,(47) | Key topics in pandemic health risk communication: A qualitative study of expert opinions and knowledge. |
| Copeland et al,(48) | A resilience view on health system resilience: a scoping review of empirical studies and reviews. |
| ECDC(49) | The EU experience in the first phase of COVID-19: implications for measuring preparedness. |
| ECDC(50) | Long-term qualitative scenarios and considerations of their implications for preparedness and response to the COVID-19 pandemic in the EU/EEA. |
| ECDC(51) | Conducting after-action reviews of the public health response to COVID-19: update. |
| ECDC(52) | The Swedish advice-making process for distance learning in schools, November 2020 − April 2021. |
| Eerens et al,(53) | The architecture of the European Union’s pandemic preparedness and response policy framework. |
| Elnaeim et al,(54) | Global and regional governance of One Health and implications for global health security. |
| European Commission(55) | HERA work plan. |
| European Parliament(56) | Regulation 2022/2371 on serious cross-border threats to health and repealing Decision No 1082/2013/EU. |
| Ewert et al,(57) | Any lessons to learn? Pathways and impasses towards health system resilience in post-pandemic times. |
| Gromek et al,(58) | Modern technologies in enhancing situational awareness and preparedness for CBRN events in urban areas. |
| Kokki et al,(59) | Preparing Europe for future health threats and crises - key elements of the European Centre for Disease Prevention and Control's reinforced mandate. |
| Lal et al,(60) | Pandemic preparedness and response: exploring the role of universal health coverage within the global health security architecture. |
| Lee et al,(61) | Public health emergency preparedness for infectious disease emergencies: a scoping review of recent evidence. |
| Li et al,(62) | Preparedness with a system integrating inventory, capacity, and capability for future pandemics and other disasters. |
| McCoy et al,(63) | Global health security and the health-security nexus: principles, politics and praxis. |
| Naik et al,(64) | Public Health Emergency Preparedness: Qualitative Analysis of After-Action Reports \| Disaster Medicine and Public Health Preparedness. |

**Table S1 Peer reviewed and grey literature included in the literature review (contd)**

| **Organisation/Country/Author** | **Title** |
| --- | --- |
| Naughton et al,(65) | Lessons from inter-disciplinary collaboration to mitigate SARS-CoV-2 transmission in schools, Ireland, 2020/2021, to inform health systems and multisectoral recovery. |
| Nazari et al,(66) | Structural Elements and Requirements in Forming Prehospital Health Response Teams in Response to Chemical, Biological, Radiation, and Nuclear Incidents (CBRN), a Comparative Review Study. |
| Phuong et al,(67) | Toward informatics-enabled preparedness for natural hazards to minimize health impacts of climate change. |
| SHARP Joint Action(68) | Strengthened International Health Regulations & Preparedness in the EU. |
| The Lancet Global Health(69) | The future of the International Health Regulations. |
| Traore et al,(70) | How prepared is the world? Identifying weaknesses in existing assessment frameworks for global health security through a One Health approach. |
| WHO(71) | Update to requirements for reporting COVID-19 surveillance data under the International Health Regulations (IHR 2005). |
| WHO(72) | Strengthening WHO preparedness for and response to health emergencies. |
| WHO(73) | Preparedness and resilience for emerging threats Module 1: planning for respiratory pathogen pandemics. |
| WHO(74) | WHO benchmarks for strengthening health emergency capacities. |
| WHO(75) | Report of the review committee regarding amendments to the IHR (2005). |
| Winters et al,(76) | Considerations of Medical Preparedness to Assess and Treat Various Populations During a Radiation Public Health Emergency. |
| Wouters et al,(77) | The launch of the EU Health Emergency Preparedness and Response Authority (HERA): Improving global pandemic preparedness? |
| Zawadzki et al,(78) | A framework for supporting health capability‐based planning: Identifying and structuring health capabilities. |

**Table S2: Guiding principles for the development of a health threats framework**

| **Guiding principles** | |
| --- | --- |
| 1. | An all hazards, One Health, whole of government and whole of society approach to health threats preparedness planning |
| 2. | Alignment with and integration into other national plans, strategies, and policies |
| 3. | Alignment with international legislation with European and global orientation to health threats preparedness, particularly for cross-border threats |
| 4. | Strong partnerships, multisectoral and intersectoral collaboration, including with community partners, agencies, organisations and representatives |
| 5. | Risk assessments using information on existing and emerging hazards based on the country risk profile and risk categorisation of the threat |
| 6. | Underpinned by adaptable, flexible, and interoperable surveillance and early warning systems |
| 7. | Flexible, adaptable and responsive strategy as health threats emerge and evolve |
| 8. | Supported by clear leadership and governance structures |
| 9. | Non-discriminatory and equitable in approach, considering and addressing underlying structural inequities and focusing on vulnerable groups |
| 10. | Adequate resourcing (financial and human) to allow implementation |
| 11. | Focus on continuous quality improvement and ongoing monitoring and evaluation |

**References**

1. Ario AR, Makumbi I, Bulage L, Kyazze S, Kayiwa J, Wetaka MM, et al. The logic model for Uganda's health sector preparedness for public health threats and emergencies. Global health action. 2019;12(1):1664103.

2. The Australian Government Department of Health. Australia's National Action Plan for Health Security 2019-2023. Australia: The Australian Government Department of Health; 2018.

3. Centers for Disease Control and Prevention. CDC Global Health Strategy 2019-2021. USA: CDC; 2019.

4. Deloitte Centre for Health Solutions. Bridging the gap: Protecting the nation from public health threats. London: Deloitte Centre for Health Solutions; 2021.

5. European Centre for Disease Prevention and Control. HEPSA – health emergency preparedness self-assessment tool. Stockholm: ECDC; 2018.

6. European Centre for Disease Prevention and Control. Public health emergency preparedness – Core competencies for EU Member States. Stockholm: ECDC; 2017.

7. European Centre for Disease Prevention and Control. Health emergency preparedness for imported cases of high-consequence infectious diseases. Stockholm: ECDC; 2019.

8. European Centre for Disease Prevention and Control. ECDC Strategy 2021–2027. Stockholm: ECDC; 2021.

9. European Centre for Disease Prevention and Control. ECDC tool for the prioritisation of infectious disease threats – Handbook and manual. Stockholm: ECDC; 2017.

10. European Centre for Disease Prevention and Control. Community engagement for public health events caused by communicable disease threats in the EU/EEA. Stockholm: ECDC; 2020.

11. European Centre for Disease Prevention and Control. A literature review on community and institutional emergency preparedness synergies. Stockholm: ECDC; 2017.

12. European Medicines Agency. EMA plan for emerging health threats. United Kingdom: EMA; 2018.

13. Department of Health and Social Care. Transforming rhe public health system: reforming the public health system for the challenge of our times. United Kingdom: Department of Health and Social Care; 2022.

14. Public Health England. PHE Infectious Diseases Strategy 2020-2025. England: Public Health England; 2019.

15. Public Health England. PHE Strategy 2020-25. England: Public Health England; 2019.

16. European Commission. Introducing HERA, the European Health Emergency preparedness and Response Authority, the next step towards completing the European Health Union. Brussels: European Commission; 2021.

17. European Commission. Action Plan to enhance preparedness against chemical, biological, radiological and nuclear security risks. Brussels: European Commission; 2017.

18. European Parliament. EU preparedness and responses to Chemical, Biological, Radiological and Nuclear (CBRN) threats. Brussels: European Parliament; 2021.

19. European Parliament. Member States' Preparedness for CBRN Threats. Brussels: European Parliament; 2018.

20. European Parliament. Regulation (EU) 2021/22 of the European Parliament and of the Council establishing a Programme for the Union's action in the field of health (EU4Health Programme) for the period 2021-2027. Brussels: European Parliament; 2021.

21. Gontariuk M, Krafft T, Rehbock C, Townend D, Van der Auwermeulen L, Pilot E. The European Union and Public Health Emergencies: Expert Opinions on the Management of the First Wave of the COVID-19 Pandemic and Suggestions for Future Emergencies. Frontiers in public health. 2021;9.

22. Welsh Government. The Communicable Disease Outbreak Plan for Wales. Wales: Government of Wales; 2020.

23. Johns Hopkins Center for Health Security. Preparedness for a High-Impact Respiratory Pathogen Pandemic. Maryland: Johns Hopkins Center for Health Security; 2019.

24. Khan Y, O’Sullivan T, Brown A, Tracey S, Gibson J, Généreux M, et al. Public health emergency preparedness: a framework to promote resilience. BMC Public Health. 2018;18(1):1344.

25. European Observatory on Health Systems and Policies. Drawing light from the pandemic: a new strategy for health and sustainable development. Copenhagen: World Health Organization. Regional Office for Europe; 2021 2021-09-10.

26. Rose DA, Murthy S, Brooks J, Bryant J. The Evolution of Public Health Emergency Management as a Field of Practice. American journal of public health. 2017;107(S2):S126-S33.

27. Sharp A, Jain V, Alimi Y, Bausch DG. Policy and planning for large epidemics and pandemics – challenges and lessons learned from COVID-19. Current Opinion in Infectious Diseases. 2021;34(5):393-400.

28. Stoto MA, Nelson C, Savoia E, Ljungqvist I, Ciotti M. A Public Health Preparedness Logic Model: Assessing Preparedness for Cross-border Threats in the European Region. Health Secur. 2017;15(5):473-82.

29. The independent panel for pandemic preparedness and response. COVID-19: make it the last pandemic. 2021.

30. Department of Health and Human Services. National Health Security Strategy 2019-2022. USA: Department of Health and Human Services; 2019.

31. Department of Health and Human Services. Strategic Plan for 2020-2023. USA: Department of Health and Human Services; 2020.

32. World Health Organization. WHO Guidance on Preparing for National Response to Health Emergencies and Disasters. Geneva: WHO; 2021.

33. World Health Organization. A Strategic Framework for Emergency Preparedness. Geneva: World Health Organisation; 2017.

34. World Health Organization. Action plan to improve public health preparedness and response in the WHO European Region 2018–2023. Denmark: WHO; 2019.

35. World Health Organization. Regional Office for the Western P. Asia Pacific strategy for emerging diseases and public health emergencies (APSED III) : advancing implementation of the International Health Regulations (2005) : working together towards health security. Manila: WHO Regional Office for the Western Pacific; 2017.

36. World Health Organization. Strengthening pandemic preparedness planning for respiratory pathogens. Geneva: WHO; 2022.

37. World Health Organization. Emergency Response Framework Second Edition. Geneva: WHO; 2017.

38. World Health Organization. International Health Regulations Monitoring and Evaluation Framework. Geneva: WHO; 2018.

39. World Health Organization. NAPHS for All. A 3 Step Strategic Framework for National Action Plan for Health Security. Geneva: WHO; 2018.

40. World Health Organization. NAPHS for all: A country implementation guide for national action plan for health security (NAPHS). Geneva: WHO; 2019.

41. World Health Organization. Strategic Preparedness, Readiness and Response Plan to End the Global COVID-19 Emergency in 2022. Geneva: WHO; 2022.

42. World Health Organization. Report of the review committee in the functioning of the International Health Regulations (2005) during the COVID-19 response. Geneva: WHO; 2021.

43. World Health Organization. International Health Regulations (2005) - 3rd Edition. Geneva: WHO; 2016.

44. World Health Organization. Global genomic surveillance strategy for pathogens with pandemic and epidemic potential, 2022–2032. Geneva: WHO; 2022.

45. Wijesinghe PR, Ofrin RH, Bhola AK, Inbanathan FY, Bezbaruah S. Pandemic influenza preparedness in the WHO South-East Asia Region: a model for planning regional preparedness for other priority high-threat pathogens. WHO South-East Asia journal of public health. 2020;9(1):43-9.

46. Alakija A. Leveraging lessons from the COVID-19 pandemic to strengthen low-income and middle-income country preparedness for future global health threats. The Lancet Infectious Diseases. 2023;23(8):e310-e7.

47. Berg SH, Shortt MT, Røislien J, Lungu DA, Thune H, Wiig S. Key topics in pandemic health risk communication: A qualitative study of expert opinions and knowledge. PloS one. 2022;17(9):e0275316.

48. Copeland S, Hinrichs-Krapels S, Fecondo F, Santizo ER, Bal R, Comes T. A resilience view on health system resilience: a scoping review of empirical studies and reviews. BMC Health Services Research. 2023;23(1):1297.

49. European Centre for Disease Prevention and Control. The EU experience in the first phase of COVID-19: implications for measuring preparedness. Stockholm: ECDC; 2022.

50. European Centre for Disease Prevention and Control. Long-term qualitative scenarios and considerations of their implications for preparedness and response to the COVID-19 pandemic in the EU/EEA. Stockholm: ECDC; 2022.

51. European Centre for Disease Prevention and Control. Conducting in-action and after-action reviews of the public health response to COVID-19: update. Stockholm: ECDC; 2023.

52. European Centre for Disease Prevention and Control. The Swedish advice-making process for distance learning in schools, November 2020−April 2021. An after-action review. Stockholm: ECDC; 2023.

53. Eerens D, Hrzic R, Clemens T. The architecture of the European Union’s pandemic preparedness and response policy framework. European Journal of Public Health. 2022;33(1):42-8.

54. Elnaiem A, Mohamed-Ahmed O, Zumla A, Mecaskey J, Charron N, Abakar MF, et al. Global and regional governance of One Health and implications for global health security. The Lancet. 2023;401(10377):688-704.

55. The European Commission. 2024 HERA Work Plan. Brussels: European Commission; 2024.

56. European Parliament and Council of the European Union. Regulation (EU) 2022/2371 of the European Parliament and the Council of 23 November 2022 on serious cross-border threats to health and repealing Decision No 1081/2013/EU. Luxembourg: Official Journal of the European Union; 2022.

57. Ewert B, Wallenburg I, Winblad U, Bal R. Any lessons to learn? Pathways and impasses towards health system resilience in post-pandemic times. Health Economics, Policy and Law. 2022;18(1):66-81.

58. Gromek P, Szklarski Ł. Modern technologies in enhancing situational awareness and preparedness for CBRN events in urban areas. Perspective of European Commission call in 2022. Journal of Modern Science. 2023;53(4):362-90.

59. Kokki M, Ammon A. Preparing Europe for future health threats and crises - key elements of the European Centre for Disease Prevention and Control's reinforced mandate. Euro surveillance : bulletin Europeen sur les maladies transmissibles = European communicable disease bulletin. 2023;28(3).

60. Lal A, Abdalla SM, Chattu VK, Erondu NA, Lee T-L, Singh S, et al. Pandemic preparedness and response: exploring the role of universal health coverage within the global health security architecture. The Lancet Global Health. 2022;10(11):e1675-e83.

61. Lee JM, Jansen R, Sanderson KE, Guerra F, Keller-Olaman S, Murti M, et al. Public health emergency preparedness for infectious disease emergencies: a scoping review of recent evidence. BMC Public Health. 2023;23(1):420.

62. Li MK, Sodhi MS, Tang CS, Yu JJ. Preparedness with a system integrating inventory, capacity, and capability for future pandemics and other disasters. Production and Operations Management. 2023;32(2):564-83.

63. McCoy D, Roberts S, Daoudi S, Kennedy J. Global health security and the health-security nexus: principles, politics and praxis. BMJ Global Health. 2023;8(9):e013067.

64. Naik R, Maxwell N, Jones T, Dopson SA. Public Health Emergency Preparedness: Qualitative Analysis of After-Action Reports. Disaster Medicine and Public Health Preparedness. 2023;17:e523.

65. Naughton P, Kelly C, White P, Kennedy E, Healy A, Collins A, et al. Lessons from inter-disciplinary collaboration to mitigate SARS-CoV-2 transmission in schools, Ireland, 2020/2021, to inform health systems and multisectoral recovery. Frontiers in public health. 2022;10:1072566.

66. Nazari S, Sharififar S, Ahmadi Marzaleh M, Zargar S, Azarmi S, Akbari Shahrestanaki Y. Structural Elements and Requirements in Forming Prehospital Health Response Teams in Response to Chemical, Biological, Radiation, and Nuclear Incidents (CBRN), a Comparative Review Study. Disaster Medicine and Public Health Preparedness. 2023;17:e300.

67. Phuong J, Riches NO, Calzoni L, Datta G, Duran D, Lin AY, et al. Toward informatics-enabled preparedness for natural hazards to minimize health impacts of climate change. Journal of the American Medical Informatics Association. 2022;29(12):2161-7.

68. Strengthened International Health Regulations & Preparedness in the EU. Monitoring and evaluation of IHR (2005) core capacities and implementation of Decision 1082/2013/EU at the European level. SHARP Joint action; 2023.

69. The Lancet Global Health. The future of the International Health Regulations. The Lancet Global Health. 2022;10(7):e927.

70. Traore T, Shanks S, Haider N, Ahmed K, Jain V, Rüegg SR, et al. How prepared is the world? Identifying weaknesses in existing assessment frameworks for global health security through a One Health approach. The Lancet. 2023;401(10377):673-87.

71. World Health Organization. Update to requirements for reporting COVID-19 surveillance data under the International Health Regulations (IHR 2005). Geneva: WHO; 2023.

72. World Health Organization. Strengthening WHO preparedness for and response to health emergencies. Geneva: WHO; 2023.

73. World Health Organization. Preparedness and resilience for emerging threats module 1: planning for respiratory pathogen pandemics. Geneva: WHO; 2023.

74. World Health Organization. WHO benchmarks for strengthening health emergency capacities. Geneva: WHO; 2023.

75. World Health Organization. Report of the Review Committee regarding amendments to the International Health Regulations (2005). Geneva: WHO; 2023.

76. Winters TA, Cassatt DR, Harrison-Peters JR, Hollingsworth BA, Rios CI, Satyamitra MM, et al. Considerations of Medical Preparedness to Assess and Treat Various Populations During a Radiation Public Health Emergency. Radiation Research. 2023;199(3):301-18.

77. Wouters OJ, Forman R, Anderson M, Mossialos E, McKee M. The launch of the EU Health Emergency Preparedness and Response Authority (HERA): Improving global pandemic preparedness? Health policy (Amsterdam, Netherlands). 2023;133:104844.

78. Zawadzki M, Montibeller G. A framework for supporting health capability-based planning: Identifying and structuring health capabilities. Risk Analysis. 2023;43(1):78-96.
